# Buruli-Ulcer Induced Disability in Ghana: A Study at Apromase in the Ashanti Region

**DOI:** 10.1155/2012/752749

**Published:** 2012-05-15

**Authors:** Pius Agbenorku, Anthony Edusei, Margaret Agbenorku, Thomas Diby, Esenam Nyador, Geoffrey Nyamuame, Paul Saunderson

**Affiliations:** ^1^Reconstructive Plastic Surgery and Burns Unit, Komfo Anokye Teaching Hospital, School of Medical Sciences, Kwame Nkrumah University of Science and Technology, Kumasi, Ghana; ^2^Department of Community Health, School of Medical Sciences, Kwame Nkrumah University of Science and Technology, Kumasi, Ghana; ^3^Health Education Unit, Global Evangelical Mission Hospital, Apromase-Ashanti, Ghana; ^4^Department of Anatomy, School of Medical Sciences, Kwame Nkrumah University of Science and Technology, Kumasi, Ghana; ^5^Centre for Disability and Rehabilitation Studies, School of Medical Sciences, Kwame Nkrumah University of Science and Technology, Kumasi, Ghana; ^6^Department of Surgery, Volta Regional Hospital, Ho, Ghana; ^7^American Leprosy Missions, Greenville, SC 29601, USA

## Abstract

*Objectives*. To describe trends and category of disabilities caused by Buruli ulcer disease. *Design*. This retrospective study was set up to quantify information on the disability trends caused by Buruli ulcer (BU) using data on patients attending BU and chronic ulcer clinics from 2004 to 2009, at Global Evangelical Mission Hospital, Apromase. *Methods*. Data was retrieved from the WHO BU1 form, case registry book, surgical theatre register, and BU patients' records book of the hospital. Disability was measured as the incapability of patients to perform one or more daily activities due to his/her state of BU disease before treatment. *Results*. A total of 336 positive BU cases comprising 181 males (53.9%) were recorded of which 113 (33.6%) cases of disabilities were identified. A mean age of 52.5 (±1.32) years was recorded. For the trend of disabilities, the year 2009 recorded the highest (*N* = 34, 31.0%). The lesions were mostly located at the lower limbs (*N* = 65, 57.5%) region of the patients. Lesions with diameter >15 cm were the major (59.3%) category of lesions. *Conclusion*. Trend of disability reveals proportional increase over the years from 2004 to 2009. Contracture at the knee and ankle joints was the commonest disability recorded.

## 1. Introduction

Buruli ulcer (BU) is an infectious disease caused by *Mycobacterium ulcerans *(MU), affecting the skin, subcutaneous tissue, and sometimes the bone. The natural reservoir of the bacillus and the mode of transmission of the disease is unclear [1, 2]. MU has been identified by molecular tools from the environment and, recently, cultured; it is generally believed to be an infection by an environmental microorganism [3]. Many different animal species appear to test positive in endemic areas, [4–7] although, a typical vector has not been convincingly identified [8, 9]. Aquatic insects, notably, *Naucoridae *spp. may serve as a vector of MU [6]. Case control studies among people living in endemic areas have identified risk factors to contract the disease; there is a striking association with stagnant and slowly flowing water bodies [10–12]. This disease has emerged dramatically in West Africa (Côte d'Ivoire, Ghana, and Benin). Prevalence rates in endemic districts in Ghana are reported to be up to 150 per 100,000 persons [13, 14].

According to the clinical case definition of the World Health Organization (WHO), the preulcerative stage includes nodules, plaques, or edema [2]. Few patients may visit a hospital with this stage of the disease. The most frequent lesion is an ulcer. In the ulcerative stage, skin ulcers with typically undermined edges can be clinically diagnosed from other skin disorders. Later, a granulomatous healing response occurs, and fibrosis, scarring, calcification, and contractures with permanent disabilities may result [15]. In rural Africa, patients tend to report to the hospital late in the course of the disease, and some long-standing and extensive lesions that have affected joints and bone may have advanced to a stage that amputation is the only reasonable option for these extensive lesions [16]. Indeed, with increasing patient delay, lesions tend to become larger resulting in more severe functional limitations [17]. In Benin, focus group discussions revealed that people believe that this disease may be natural, “sent directly by God,” or induced by another person through sorcery [18]. Most interviewees admitted that they would first see a traditional healer before considering treatment in a hospital. In Ghana, most healthy respondents (without BU) said they would attend the hospital with an ulcerative lesion but would try herbal treatment if the lesion were a nodule; patients with BU had admitted that they would only go to the hospital if lesions became larger than expected [19]. Fear of surgery might be one of the factors associated with patients' decision to postpone having treatment in a hospital [1]. 

Antibiotic treatment alone was successful in achieving a cure in many patients, and it requires daily injections with Streptomycin (10 mg/kg body weight) and oral Rifampicin, 15 mg/kg body weight. Also, a combination of chemotherapy and surgical treatment had been reported to be effective in the treatment of BU. Techniques used in surgical treatment consist generally of two stages. In the first stage the aim is to excise all dead tissue, including a healthy tissue margin around the lesion—typically 3-4 cm [13]. This procedure results in a large surgical lesion that needs to be skin grafted in a second stage. Surgery involving the head and neck region, especially the face and eyes, poses difficult reconstructive plastic surgical problems for surgeons [20–22]. Surgery is difficult to perform in settings with poor resources, as there are very limited possibilities for general anesthesia and blood transfusion [1]. Moreover, it requires extensive technical and surgical skills, and the patient will undergo several months of wound dressing, hence some expenses will be incurred by the patient for the dressing. Finally, with such large excisions, the wounds eventually heal at the expense of more severe sequelae than those resulting from less extensive surgery, conceivably resulting in more disabilities, especially if no form of physical therapy is given to prevent contractures.

Antibiotic treatment with Streptomycin is not an option for pregnant women who are treated by surgery. Physical disabilities due to scar contracture may result in psychosocial and economic problems as reported by some studies [19, 23, 24]. However, Lehman et al. [25] reported that the 6th WHO Advisory Committee on Buruli ulcer recommended directly observed treatment with the combination of Rifampicin and Streptomycin, administered daily for 8 weeks. The study of Chauty et al. [26] concludes that the WHO-recommended Streptomycin-Rifampicin combination is highly efficacious for treating M. ulcerans disease.

This retrospective study was carried out to describe the trends and category of the disability and the body parts mostly involved in deformities caused by BU disease among patients who visited Global Evangelical Mission Hospital (GEMH) at Apromase in the Ejisu-Juaben Municipal, Ashanti Region, Ghana, from 2004 to 2009.

## 2. Materials and Methods

### 2.1. Study Setting

Ejisu-Juaben municipal stretches over an area of 637.2 km^2^ constituting about 10% of the entire Ashanti Region and with Ejisu as its capital. The Municipal is located in the central part of the Ashanti Region. Patients come from the entire surrounding districts to seek BU treatment at GEMH.

The population of the Municipality is about 120,896, with a male to female ratio of 1 : 08. Children (0–14 years) and individuals aged over 60 years form about 16.5 and 5.6 percent of the total population, respectively. The municipal is divided into five zones, namely, Ejisu, Juaben, Achiasi, Bomfa and Kwaso submunicipals. Bomfa (which is a town in the Bomfa submunicipal) is a BU disease endemic area [17, 27] located about 36 km southeast of Ejisu. It is also about 10 km southwest of Konongo, which is the district capital of the nearby Ashanti-Akim North District. There are four primary health care facilities in Bomfa sub-municipal and its environs, namely Bomfa Health Centre at Bomfa, Agyenkwa Clinic at Hwereso, the Adventist (SDA) Clinic at Nobewam, and Huttel Health Centre at Buamadumase; these facilities do not yet have the infrastructure to diagnose and treat BU and so refer their BU patients to GEMH, which is in the nearby Ejisu submunicipal.

### 2.2. Treatment Center

The Global Evangelical Mission Hospital is located at Apromase in the Ejisu submunicipal, a village about 10 km southwest of Ejisu and also about 12 km southeast of Kumasi. The hospital has thirty-five beds. It is owned by the Global Evangelical Church with its headquarters in Accra, the capital city of Ghana.

### 2.3. Data Acquisition

Disability was measured in the study as the incapability of patients to perform one or more daily activities due to his/her state of BU disease before treatment at GEMH. Thus, patients whose stage of BU disease when reported for treatment at GEMH had affected their daily activities were considered as disabled in the study.

BU cases were diagnosed in this study by the confirmation by any two positive of Ziehl-Neelson (ZN) test for acid fast bacilli, polymerase chain reaction (PCR), and histopathology. BU patients whose records fitted in the category of the study were selected.

Information on patients attending BU and chronic ulcer clinics at GEMH from 2004 to 2009 was utilized for the study. Ethical approval for the study was obtained from the Ethical Committee of the School of Medical Sciences, Kwame Nkrumah University of Science and Technology, Kumasi. Data for the study was retrieved from the WHO BU1 forms, case registry book, surgical theater register, and BU patients' records book of the hospital. Data obtained include demographic features of patients, laboratory investigations, and category and location of lesion(s). Data obtained was recorded, analyzed, and displayed in tables and graphs using SPSS version 16.0 (SPSS, Inc., Chicago, IL, USA).

### 2.4. Clinical Diagnosis

Diagnosis of BU was based on clinical findings such as chronicity of the wound, typical undermined edges with central necrotic tissues, and failure to respond to traditional wound management procedures and antibiotic therapy. The findings were then confirmed by any two positive of ZN test for acid fast bacilli, PCR, and histopathology.

### 2.5. Patients Management

According to current WHO guidelines, BU treatment should be surgery combined with chemotherapy (a combination of Rifampicin and Streptomycin/Amikacin for 8 weeks as a first-line treatment) and physiotherapy [25, 27]. All patients had antibiotics (Streptomycin and Rifampicin for 8 weeks); patients without contraindication for antibiotic treatment (e.g., pregnancy) were treated with Streptomycin and Rifampicin daily. Patients with ulcers had debridement and skin grafting. Some of them, because of the location of the ulcers (immediately adjacent to the eye), could not have complete excision. They were dressed over long periods with normal saline or 2% acetic acid lotions. Because of the difficulty in achieving good hemostasis, some of the excised ulcers were grafted secondarily within 48–72 hours or a week later. Some recurrent ulcerated cases at different anatomic parts of the body were confirmed positive by both ZN and PCR techniques. There was no sign of antibiotic resistance, and the lesions were treated with the combination of surgery and chemotherapy.

## 3. Results

Information on 864 patients was retrieved, out of which 336 positive BU cases composed of more males (*N* = 181, 53.9%) than females (*N* = 155, 46.1%) were recorded during the study period. All stages (preulcer, ulcer, and healing stages) of the disease were identified, with the ulcer stage being the dominant (*N* = 248, 73.8%) followed by healing stage (*N* = 79, 23.5%). It was also noted that all the patients who had a disability were those from the ulcer stage. Again, the majority (62.2%) of the study patients resided in the Ejisu-Juaben Municipality.

**Figure 1:**
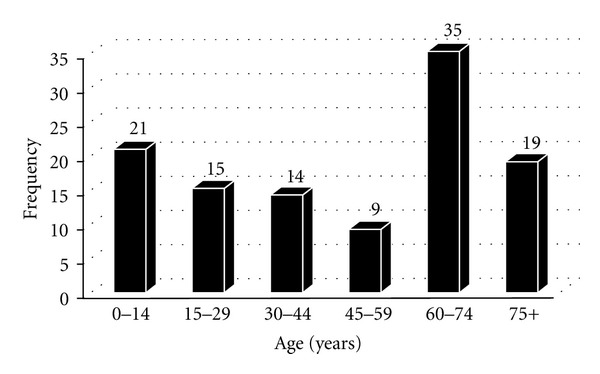
Age distribution of BU functional disabilities patients (*N* = 113).

In terms of disabilities, out of the 336 positive BU patients who patronized the facility, 113 developed disabilities at different parts of their bodies. The ages of the BU disabled patients cut across all the age groups (a mean age of 52.5 (±1.32) years), with the bulk of them falling within 60–74 years followed by 0–14 years old children (Figure 1).

Each patient was taken through the three laboratory investigations. The laboratory report (ZN, culture, PCR, and histopathology) of the 336 patients revealed that PCR and histopathology recorded the highest sensitivity as compared to the other tests.  Table 1 shows the age groups of patients against the number of BU patients who tested positive in each laboratory investigation.

**Figure 2:**
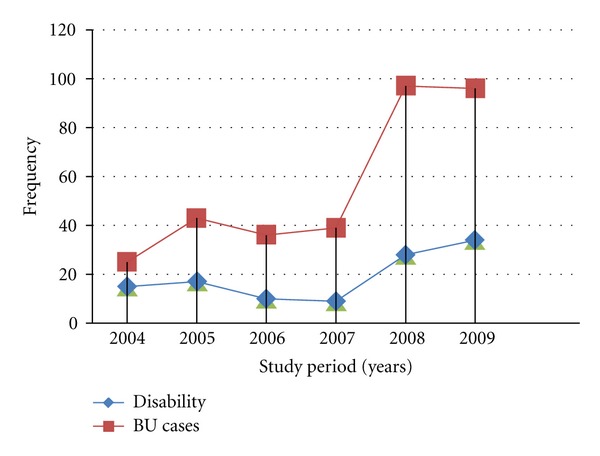
Trend of Buruli Ulcer cases and functional disabilities.

The trend of BU cases and disabilities was found to be increasing from 2004 to 2009, with the year 2008 having the highest BU cases (*N* = 97, 28.9%). In terms of percentage disability, 2007 recorded 22.5% and 2008 recorded 29.9%, while 2009 registered 31.0% (Figure 2).

The contractures were mostly located at the lower limbs (*N* = 65, 57.5%), with few (6.2%) located in the head and neck region (HNR) of the patients (Table 2).

**Figure 3:**
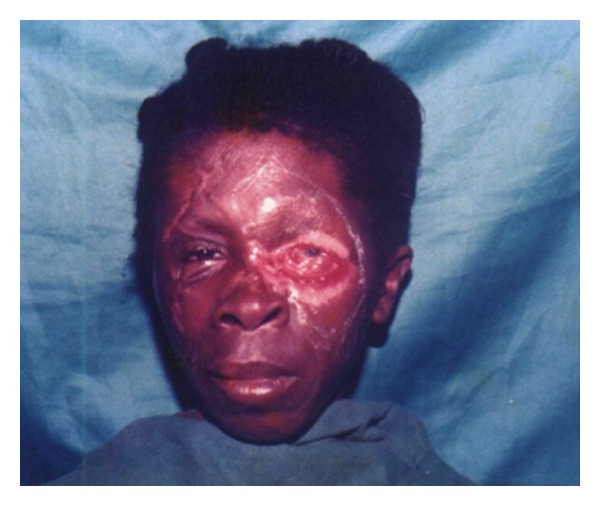
Buruli Ulcer Sequelae of the face.

**Figure 4:**
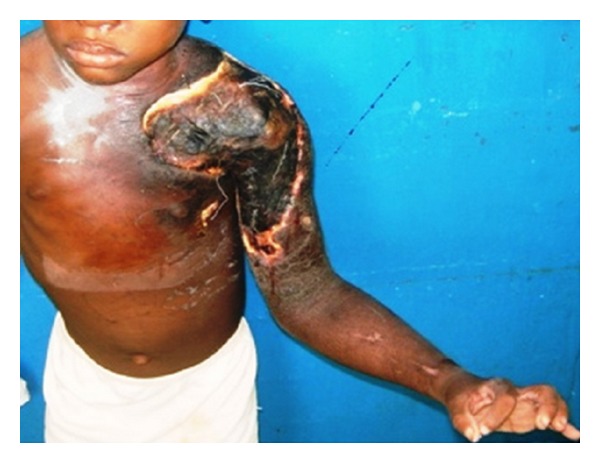
Shoulder joint contracture caused by Buruli ulcer.

**Figure 5:**
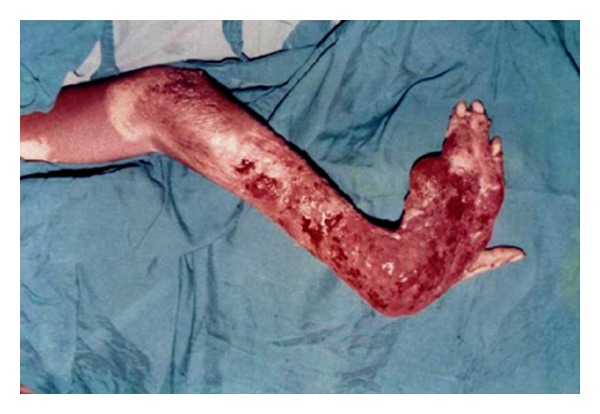
Wrist contracture of the left hand caused by Buruli ulcer.

Representations of these locations are shown in Figures 3, 4, and 5.

**Figure 6:**
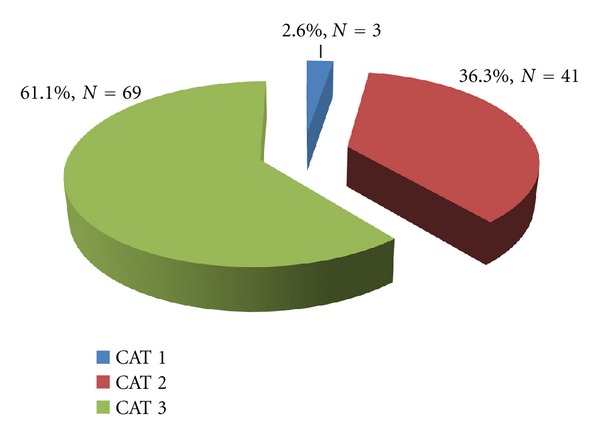
A chart showing the distribution of the categories of lesions.

The category of lesions had been classified into three (3) groups according to WHO [25]. Category (CAT) 1 is for single lesion <5 cm in diameter, CAT 2 for single lesion 5–15 cm in diameter, and CAT 3 > 15 cm for single or multiple lesions at critical sites such as the joints and others may be osteomyelitis. The CAT 3 lesions were identified to contribute mostly (*N* = 69, 61.1%) in the BU disability development (Figure 6).

## 4. Discussion

In our study, patients who developed sequelae were those who do not come to the hospital until the ulcerative stage of disease. Out of the 336 positive BU cases 113 (33.6%) developed disabilities. A similar study in Côte D'Ivoire recorded 82 (26.0%) of patients with healed ulcers who had chronic disability [27]. Age is an important demographic factor in terms of BUD, and a lot of studies had shown that children (0–14 years) are the most vulnerable, while gender is insignificant [27]. However, concerning disabilities, Stienstra et al. (2005) reported that, increased age and female sex were independent risk factors for functional limitation [28]. The results of our study reveal that most of the BU disabled individuals were adults (Figure 1). Our findings buttress that of Stienstra et al. (2005), and the reason for this alternation in age groups in terms of BU infection and disability may be ignorant of the preulcerative stage of the disease by most adults, believing that this disease may be natural, “sent directly by God,” or induced by another person through sorcery [18]. Trying herbal treatment [29] and the fear of surgery might be one of the factors associated with patients' decision to postpone having treatment in a hospital [1].

A combination of Rifampicin and Streptomycin administered daily for 8 weeks had been reported to be very effective in the management of BUD [25]. However, in this study, few patients had a recurrent BUD. The study of O'Brien et al. [30] and that of Ruf et al. [31] reveal that most lesions appearing during or after antibiotics treatment are due to paradoxical reaction, and this will be the case in our study, since the cultured specimen recorded no growth, despite ZN and PCR being positive.

Buruli ulcer disabilities were also identified to have a relatively direct increasing pattern with the study years (Figure 2). This trend which is illustrated graphically may have been influenced by factors such as health education and popularity of GEMH in terms of management of BU patients. The hospital started treatment of BU cases in July 2004, this is revealed in the least number of cases recorded (Figure 2). Although the BU cases were minimal, a relatively high number (60%) of them developed disabilities. The following three years (i.e., 2005–2007) were backed by a strong health education team to create awareness and educate the public on BUD in the district and environs. This health education program contributed a lot; patients who had been rejected by their families due to the belief that they had been cursed and others with long futile herbal medications had hope at last. This situation faced by the BU patients had been confirmed in earlier studies by Aujoulat et al. [18] and Stienstra et al. [19]. The number of BU disabilities recorded dropped, even though there was a slight increase of BU cases towards 2007. The reason for this decrease in disabilities may be as a result of the health education, since most of the cases within those years were in the preulcerative stage. This fact has already been suggested by Lehman et al. in BU prevention of disability for WHO [25]. Furthermore, the sudden escalation of both BU cases and disabilities for the other two years (i.e., 2008 and 2009) may be due to the popularity of the hospital in terms of BU treatment and patient management, since most of the patients treated within those years were patients who had been referred from different hospitals in the region and beyond with large chronic ulcers (Figure 6; Table 1). Most of these patients developed various disabilities and horrible scars—leading to limited eye closure, limited extension and flexion of muscles, limited knee extension and foot dorsiflexion, deviation of the hand and foot, among others—which may also affect them psychologically and socially [25].

Earlier studies had reported that lesions at a joint are associated with increased risk for residual functional limitations [29, 32, 33]. Furthermore, amputation and visible muscular atrophy were associated with increased chance of residual functional limitations in previous studies [1, 28, 32]. Nonhealed lesions and a lesion at a distal part of an extremity were among independent risk factors for functional limitation in an earlier study [28]. Ellen et al. [24] in their assessment of functional limitation caused by BUD reported that out of 78 BU patients 58% had a reduction in the range of motion of one or more joints, 30% had one or more functional limitations of the leg and 21% of the arm, 49% had a functional limitation. The findings of our study were similar to the previously mentioned studies. Lesions at joints and the extremities of limbs were prone to disabilities due to the development of contractures or amputations (Table 2). Although disabilities such as visual impairment and psychological effects due to facial scars associated with the head and neck region of BU patients (Figure 3) were relatively few in our study, the level of disaster caused is equally severe, since the individual is incapacitated in almost all aspects of life, even in formal education especially when the victim is a child [21, 25].

Barogui et al. [1] in their study reported that a lesion >15 cm had the highest percentage of patients with functional limitation. Debacker et al. [34] in a Benin study reported that a year after the introduction of antibiotic treatment to all patients with Buruli ulcer in Benin, the number of small (category I) lesions (<5 cm) at presentation increased while the number of large (>15 cm; category III) lesions decreased. He added that “it might reflect earlier reporting, perhaps as a result of the introduction of antibiotic treatment or of patient education”. The results of this current study reveal that most of the lesions had a diameter >15 cm; lesion <5 cm were located around the eye region (Figure 6). This is practically the same as that of Barogui et al. [1], however, not in line with the report of Debacker et al. [34]. This might be due to the delay in seeking appropriate medical treatment by most of the patients, hence, report to GEMH with already deteriorated BU conditions.

As advocated by WHO in “Concepts in prevention of disability and rehabilitation”, disability in BU can be prevented or minimized with early diagnosis of the nodules, antibiotic treatment, and surgical excision—together with adequate management of the skin, soft tissues, and joints during the wound-healing process [25]. In addition it should be noted that intervention to prevent disability start before excision and continue after excision and skin grafting, in order to prevent soft tissue and joint contractures. Prevention of disability and rehabilitation are only possible with the active participation of those affected by BU, their families, the community, and the health-care team.

## 5. Conclusion

The trend of disability after the study reveals a proportional increase from 2004 to 2009. Contracture at the knee and ankle joint after healing was the highest disability recorded. Management difficulties and disabilities caused by BUD could be avoided by early detection and treatment of the disease, supported by intensive health education programs within the municipality and its environs.

## Figures and Tables

**Table 1:** Distribution of laboratory confirmation of the Buruli ulcer patients (*N* = 336).

	Laboratory test			
Age	ZN	Culture	PCR	Histopathology
0–14	33	48	63	63
15–29	21	27	45	45
30–44	21	30	42	42
45–59	18	21	27	27
60–74	57	66	102	101
75+	27	45	57	57

Total	177	237	336	335

**Table 2:** Location and distribution of Buruli ulcer sequelae on patients (*N* = 113).

Location of sequelae	Frequency (%)
Lower extremity contracture	56 (49.6)
Upper extremity contracture	38 (33.7)
Amputation within the lower extremities	9 (8.0)
Amputation within the upper extremities	3 (2.6)
Loss of eye (s)	2 (1.7)
Loss of eyelids	4 (3.5)
Loss of nose (or part of it)	1 (0.9)

## References

[1] Barogui Y, Johnson RC, van der Werf TS (2009). Functional limitations after surgical or antibiotic treatment for Buruli ulcer in Benin. *American Journal of Tropical Medicine and Hygiene*.

[2] Stienstra Y, Dijkstra PU, GuédéNon A (2004). Development of a questionnaire assessing Buruli ulcer-induced functional limitation. *American Journal of Tropical Medicine and Hygiene*.

[3] Portaels F, Meyers WM, Ablordey A (2008). First cultivation and characterization of *Mycobacterium ulcerans* from the environment. *PLoS Neglected Tropical Diseases*.

[4] Durnez L, Eddyani M, Mgode GF (2008). First detection of mycobacteria in African rodents and insectivores, using stratified pool screening. *Applied and Environmental Microbiology*.

[5] Johnson PDR, Azuolas J, Lavender CJ (2007). *Mycobacterium ulcerans* in mosquitoes captured during outbreak of Buruli ulcer, SouthEastern Australia. *Emerging Infectious Diseases*.

[6] Marsollier L, Robert R, Aubry J (2002). Aquatic insects as a vector for *Mycobacterium ulcerans*. *Applied and Environmental Microbiology*.

[7] Portaels F, Chemlal K, Elsen P (2001). *Mycobacterium ulcerans* in wild animals. *OIE Revue Scientifique et Technique*.

[8] Williamson HR, Benbow ME, Nguyen KD (2008). Distribution of *Mycobacterium ulcerans* in Buruli ulcer endemic and non-endemic aquatic sites in Ghana. *PLoS Neglected Tropical Diseases*.

[9] Benbow ME, Williamson H, Kimbirauskas R (2008). Aquatic invertebrates as unlikely vectors of Buruli ulcer disease. *Emerging Infectious Diseases*.

[10] Debacker M, Portaels F, Aguiar J (2006). Risk factors for buruli ulcer, Benin. *Emerging Infectious Diseases*.

[11] Pouillot R, Matias G, Wondje CM (2007). Risk factors for Buruli ulcer: a case control study in Cameroon. *PLoS Neglected Tropical Diseases*.

[12] Raghunathan PL, Whitney EAS, Asamoa K (2005). Risk factors for Buruli ulcer disease (*Mycobacterium ulcerans* infection): results from a case-control study in Ghana. *Clinical Infectious Diseases*.

[13] van der Werf TS, van der Graaf WTA, Tappero JW, Asiedu K (1999). *Mycobacterium ulcerans* infection. *The Lancet*.

[14] Amofah G, Bonsu F, Tetteh C (2002). Buruli ulcer in Ghana: results of a national case search. *Emerging Infectious Diseases*.

[15] Stienstra Y, van der Graaf WTA, Te Meerman GJ, The TH, de Leij LF, van der Werf TS (2001). Susceptibility to development of *Mycobacterium ulcerans* disease: review of possible risk factors. *Tropical Medicine and International Health*.

[16] van der Werf TS, Stienstra Y, Johnson RC (2005). *Mycobacterium ulcerans* disease. *Bulletin of the World Health Organization*.

[17] Agbenorku P, Agbenorku M, Bentsir-Elegba E (2011). Multicenter plastic surgery outreach services for underserved Ghanaian communities. *European Journal of Plastic Surgery*.

[18] Aujoulat I, Johnson C, Zinsou C, Guédénon A, Portaels F (2003). Psychosocial aspects of health seeking behaviours of patients with Buruli ulcer in southern Benin. *Tropical Medicine and International Health*.

[19] Stienstra Y, van der Graaf WTA, Asamoa K, van der Werf TS (2002). Beliefs and attitudes toward Buruli ulcer in Ghana. *American Journal of Tropical Medicine and Hygiene*.

[20] Agbenorku P (2000). *Mycobacterium ulcerans* Skin ulcer (MUSU) of the face. *West African Journal of Medicine*.

[21] Agbenorku P (2005). *Mycobacterium ulcerans* disease in the head and neck region. *Indian Journal Of Clinical Practice*.

[22] Aguiar J, Stenou C (1997). Les ulcères de Buruli en zone rurale au Bénin: prise en charge de 635 cas. *Medecine Tropicale*.

[23] Asiedu K, Etuaful S (1998). Socioeconomic implications of Buruli ulcer in Ghana: a three-year review. *American Journal of Tropical Medicine and Hygiene*.

[24] Ellen DE, Stienstra Y, Teelken MA, Dijkstra PU, van der Graaf WTA, van der Werf TS (2003). Assessment of functional limitations caused by *Mycobacterium ulcerans* infection: towards a Buruli ulcer functional limitation score. *Tropical Medicine and International Health*.

[25] Lehman L, Simonet V, Saunderson P, Agbenorku P (2005). *Buruli Ulcer: Prevention of Disability (POD)*.

[26] Chauty A, Ardant MF, Adeye A (2007). Promising clinical efficacy of streptomycin-rifampin combination for treatment of buruli ulcer (*Mycobacterium ulcerans* disease). *Antimicrobial Agents and Chemotherapy*.

[27] Agbenorku P, Agbenorku M, Saunderson P, Lehman L (2006). The benefits of a combination of surgery and chemotherapy in the management of Buruli ulcer patients. *Journal of Science and Technology*.

[28] Marston BJ, Diallo MO, Horsburgh CR (1995). Emergence of Buruli ulcer disease in the Daloa region of Cote d’Ivoire. *American Journal of Tropical Medicine and Hygiene*.

[29] Stienstra Y, van Roest MHG, van Wezel MJ (2005). Factors associated with functional limitations and subsequent employment or schooling in Buruli ulcer patients. *Tropical Medicine and International Health*.

[30] O’Brien DP, Robson ME, Callan PP, McDonald AH (2009). ‘Paradoxical’ immune-mediated reactions to *Mycobacterium ulcerans* during antibiotic treatment: a result of treatment success, not failure. *Medical Journal of Australia*.

[31] Ruf M-T, Chauty A, Adeye A (2011). Secondary Buruli ulcer skin lesions emerging several months after completion of chemotherapy: paradoxical reaction or evidence for immune protection?. *PLoS Neglected Tropical Diseases*.

[32] Agbenorku P, Akpaloo J, Amofa GK (2000). Sequelae of *Mycobacterium ulcerans* infections (Buruli ulcer). *European Journal of Plastic Surgery*.

[33] Teelken MA, Stienstra Y, Ellen DE (2003). Buruli ulcer: differences in treatment outcome between two centres in Ghana. *Acta Tropica*.

[34] Debacker M, Aguiar J, Steunou C (2004). *Mycobacterium ulcerans* disease (Buruli ulcer) in rural hospital, Southern Benin, 1997-2001. *Emerging Infectious Diseases*.

